# The complete chloroplast genome sequence of the *Citrullus lanatus* L. Subsp. Vulgaris (Cucurbitaceae)

**DOI:** 10.1080/23802359.2016.1261611

**Published:** 2016-12-23

**Authors:** Qianglong Zhu, Haonan Cui, Yulong Zhao, Peng Gao, Shi Liu, Pengfei Wang, Feishi Luan

**Affiliations:** a Horticulture College, Northeast Agricultural University, Harbin, Heilongjiang, China;; b Key Laboratory of Biology and Genetic Improvement of Horticultural Crops (Northeast Region), Ministry of Agriculture, Harbin, Heilongjiang, China

**Keywords:** Chloroplast genome, *Citrullus lanatus* L, cultivated watermelon

## Abstract

Watermelon is one of the worldwide popular summer fruits and well-known for colourful, sweet, and juicy flesh. The complete nucleotide sequence of cultivated watermelon (*Citrullus lanatus* L. subsp. vulgaris) chloroplast genome has been determined in this study. The genome was composed of 156,906 bp containing a pair of inverted repeats (IRs) of 26,082 bp, which was separated by a large single-copy region of 70,063 bp and a small single-copy region of 17,895 bp. A total of 114 genes were predicted included 80 protein-coding genes, four rRNA genes, and 30 tRNA genes. Phylogenetic analysis revealed that *C. lanatus* and other three species belonging to the genus *Cucumis* were closely clustered into a clade, the family Cucurbitaceae.

Watermelon is one of the worldwide popular summer fruits belonging to the family Cucurbitaceae. Its fruits are well-known for colourful, sweet, and juicy flesh. Chloroplasts are organelles mainly present in leaf cell or other green tissue of plant. They contain their own genetic system as well as the entire machinery necessary for the process of photosynthesis. Meanwhile, studies have reported that the watermelon chloroplast genome was associated with the sexual differentiation (Levi et al. 2006), and was important for plant taxonomy (Dane et al. 2004; Hu et al. 2011) and phylogenetic system researches (Dane & Lang 2004; Levi & Thomas 2005; Dane & Liu 2007; Dane et al. 2007) in watermelon.

To date, complete chloroplast genome sequences of many species in the family Cucurbitaceae have been published at NCBI Organelles Genome Database, including cultivated cucumber (Kim et al. 2006; Chung et al. 2007; Pląder et al. 2007), wild cucumber (Wu et al. 2014), melon (Rodriguez-Moreno et al. 2011). However, complete chloroplast sequence of cultivated watermelon has not yet been reported and only several partial chloroplast genome sequences were reported. In this study, we reported complete sequence of chloroplast genome of *C. lanatus* to provide basic genetic information and help understand genomic diversity of watermelon species.

The genomic DNA was extracted from young leaves of *C. lanatus* collected from and cultivated in the farm field of Northeast Agricultural University (126°43′16.7′′E, 45°44′23.8′′N), Harbin, China. A pair-end (PE) library with 300 bp insert was constructed and sequenced using an Illumina Hiseq 2000 platform by BGI, Shenzhen, China. Whole genome sequence data of 20 Gb were generated and trimmed, high quality PE reads of 0.4 Gb were randomly extracted and assembled with the BAC end sequences (Guo et al. 2013) using SPAdes (v3.6.2) (Bankevich et al. 2012), as described in Rodriguez-Moreno et al.(Rodriguez-Moreno et al. 2011). Contigs representing the chloroplast genome were retrieved, ordered and joined into a single draft sequence by comparison with the chloroplast genome of *Cucumis sativus* (GenBank accession no. NC_007144.1) as a reference (Kim et al. 2006). The draft sequence was confirmed and manually corrected by PE read mapping. The draft sequence was annotated using an integrated web server, CpGAVAS (Chang et al. 2012), and manual curation based on BLAST searches.

The complete chloroplast genome of *C. lanatus* was double-stranded circular DNA with 156,906 bp in length (GenBank accession no. KY014105). Its structure was similar to most of the described chloroplast genomes from cucurbit plants (Kim et al. 2006; Chung et al. 2007; Pląder et al. 2007; Rodriguez-Moreno et al. 2011), composed of two inverted repeated regions (IRa and IRb) of 25,150 bp, which was divided by a large single-copy (SSC) region of 70,063 bp and a small single-copy (LSC) region of 17,895 bp. The genome was typical of AT-rich, and its GC contents were 37.2%. A total of 114 genes were predicted, including 80 protein-coding genes, 30 tRNA genes, and four rRNA genes of which 17 genes were duplicated in IR regions.

Phylogenetic analysis was conducted with common 53 chloroplast protein-coding sequences of *C. lanatus* and other 11 species, using a Neighbour-Joining method of MEGA 7.0 with 1000 bootstrap replicates (Tamura et al. 2013). The phylogenetic tree indicated that *C. lanatus* and other three species belonging to the genus *Cucumis* were closely clustered into a clade, the family Cucurbitaceae, and other species belonging to same family were also well clustered into their corresponding clades, as expected (Figure 1).

**Figure 1.**
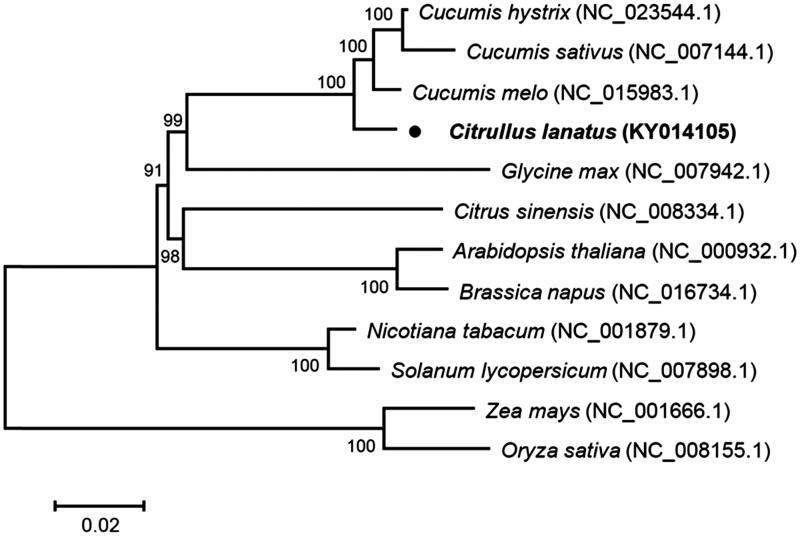
Phylogenetic tree showing relationship between *C. lanatus* and 11 species belonging to different families. Phylogenetic tree was constructed based on 53 protein-coding genes of chloroplast genomes using Neighbour-Joining method with 1000 bootstrap replicates. Numbers in each of the node indicated the bootstrap support values.
